# Complete Genome Sequence of *Curtobacterium* sp. Strain SGAir0471, Isolated from Singapore Air Samples

**DOI:** 10.1128/MRA.00960-19

**Published:** 2019-11-21

**Authors:** Phu Pwint Thin Hlaing, Ana Carolina M. Junqueira, Akira Uchida, Rikky W. Purbojati, Anthony Wong, Kavita K. Kushwaha, Alexander Putra, Carmon Kee, Nicolas E. Gaultier, Balakrishnan N. V. Premkrishnan, Cassie E. Heinle, Serene B. Y. Lim, Vineeth Kodengil Vettath, Daniela I. Drautz-Moses, Stephan C. Schuster

**Affiliations:** a Singapore Centre for Environmental Life Sciences Engineering, Nanyang Technological University, Singapore; b Departmento de Genética, Instituto de Biologia, Universidade Federal do Rio de Janeiro, Rio de Janeiro, Brazil; Indiana University, Bloomington

## Abstract

*Curtobacterium* sp. strain SGAir0471 was isolated from tropical air samples collected in Singapore. The genome was assembled using PacBio RS II long reads and Illumina MiSeq short paired-end reads. The complete genome measures 3.53 Mb and consists of 3,151 protein-coding genes, 49 tRNAs, and 12 rRNAs.

## ANNOUNCEMENT

The genus *Curtobacterium* contains rod-shaped Gram-positive bacteria and was established as a new genus in 1972 in a taxonomic study carried out on coryneform bacteria (1). *Curtobacterium* spp. mainly inhabit plants and often soil (2, 3). Some *Curtobacterium* spp. are well-established plant pathogens (4) and have also been isolated from clinical samples from patients with respiratory distress, soft tissue infection, conjunctivitis, and lymphadenopathy (5). Thus, a complete genome of a *Curtobacterium* sp. may help us to understand the possible mechanisms used by this genus to cause infections.

*Curtobacterium* sp. strain SGAir0471 was isolated from outdoor air in Singapore (1.2233N, 103.84472E) using an Andersen single-stage impactor (SKC, Inc., USA). Airborne particles were impacted onto potato dextrose agar (Sigma-Aldrich, USA), and further isolation was done on Trypticase soy agar (Becton, Dickinson, USA) at 30°C until an axenic culture was obtained. Genomic DNA was purified using the Wizard Genomic DNA purification kit (Promega, USA) following the manufacturer’s protocol. The sequencing library was prepared with the SMRTbell template prep kit 1.0 (Pacific Biosciences, USA), followed by single-molecule real-time (SMRT) sequencing conducted on the PacBio RS II platform. Additionally, short reads were generated on a MiSeq (Illumina, USA) 300-bp paired-end run using whole-genome shotgun libraries constructed with the TruSeq Nano DNA library preparation kit (Illumina, USA).

Default parameters were used for all software unless otherwise specified. A total of 44,706 subreads were used for *de novo* assembly with the Hierarchical Genome Assembly Process (HGAP) version 3 included in the PacBio SMRT Analysis version 2.3.0 package (6). Quality control of long reads was performed using the PreAssembler filter version 1 protocol from HGAP, and Cutadapt version 1.8.1 (7) was used to remove adapter sequences for short reads. Further improvements to genome quality were achieved by polishing the draft assembly via Quiver (6) and correcting errors using Pilon version 1.16 (8) using 706,319 MiSeq paired-end reads. The consensus assembly generated one chromosomal contig of 3,531,991 bp (124.2-fold coverage) with an average G+C content of 71.7%.

Genome annotation was done using NCBI’s Prokaryotic Genome Annotation Pipeline (PGAP) version 4.4 (9). The complete genome was predicted to contain a total of 3,294 genes, including 3,151 protein-coding genes (PCGs), 12 ribosomal subunits (5S, 16S, 23S), 49 tRNAs, 3 noncoding RNAs, and 79 pseudogenes.

Taxonomical identification was performed using average nucleotide identity (ANI) utilizing the Microbial Species Identifier (MiSI) method (10), which was run using ANICalculator with default parameters against a database of 6,387 bacterial RefSeq genomes created using a text filter for “type, synonym type, and proxytype” and subsequently using the getorf program with the -find 3 option. This revealed 78.4% identity with Curtobacterium ammoniigenes NBRC 101786 with an alignment fraction value of 0.22. The 16S rRNA analysis using Barrnap version 0.7 (11) and BLASTn (12) was run against the SILVA database (13) and resulted in a 99.7% identity with *Curtobacterium* sp. strain B18. As the ANI result is below the threshold for species-level identification, the *Curtobacterium* genus was assigned to the isolate based on the combined ANI and 16S rRNA sequence similarity results.

### Data availability.

The genome sequence of *Curtobacterium* sp. strain SGAir0471 has been deposited in DDBJ/EMBL/GenBank under the accession number CP027869. The SRA database accession numbers are SRR8894900 and SRR8894901.
